# CT density of cervical thymus, in comparison with mediastinal thymus

**DOI:** 10.1186/s13244-019-0781-z

**Published:** 2019-09-30

**Authors:** Anitha Sen, Jiji Valsalamony, Jubie Raj

**Affiliations:** Department of Radiodiagnosis, Regional Cancer Centre, Thiruvananthapuram, Kerala 695011 India

**Keywords:** Tomography (X-ray computed), Neck, Mediastinum

## Abstract

**Objectives:**

Cervical component of thymus is noted more in children and young adults than in older age group. CT texture (lobules of soft tissue interspersed with fat), similarity with CT density of mediastinal thymus and continuity with mediastinal thymus on sagittal/coronal images, are given as the criteria for diagnosis of the cervical thymus. But CT densities of cervical and mediastinal components of the thymus may vary. The purpose of our study was to compare CT densities of cervical and mediastinal parts of the thymus, in cases where ultrasonography correlation was available.

**Methods:**

We retrospectively identified 22 patients who had undergone CT between May 2015 and May 2017 and in whom ultrasonography (USG) correlation was available. CT densities of cervical and mediastinal components of thymus were measured.

**Results:**

1. CT density of cervical thymus is lower than the CT density of mediastinal thymus by ~ 25 HU.
2. There is a moderate positive correlation between CT densities of cervical and mediastinal parts of the thymus.
3. CT densities of both cervical and mediastinal thymus were found to reduce with age, but the reduction was statistically significant only in the cervical thymus in this study.

**Conclusions:**

- CT densities of cervical and mediastinal components of the thymus may vary, with CT density of cervical thymus being lower. There is a positive correlation between CT densities of cervical and mediastinal parts of the thymus.
- CT density of cervical thymus reduces with age.

Understanding these may help avoid confusion on CT and avoid the need for correlative USG, saving time and effort.

**Electronic supplementary material:**

The online version of this article (10.1186/s13244-019-0781-z) contains supplementary material, which is available to authorized users.

## Key points

- CT densities of cervical and mediastinal components of the thymus may vary, with CT density of cervical thymus being lower.
- CT density of cervical thymus reduces with age.
- Understanding these may help avoid confusion on CT and avoid the need for correlative USG, saving time and effort.

## CT density of cervical thymus, in comparison with mediastinal thymus

Although the thymus is predominantly a mediastinal organ, part of it can often be seen in lower neck normally [1, 2]. When seen in the lower neck, cervical thymus has to be differentiated from pathologies like cervical lymph node, thyroid/parathyroid mass, or anatomic variants like enlarged distal thoracic duct [3]. In children, branchial cleft anomaly, teratoma, venous malformation, and lipoma also need to be excluded [4]. CT texture (lobules of soft tissue interspersed with fat) [1], similarity with CT density of mediastinal thymus [5] and continuity with mediastinal thymus on sagittal/coronal images, are given as the criteria for diagnosis of normal cervical thymus. The unique ultrasound appearance of thymic tissue (echogenic linear structures/foci in a hypoechoiec background) also allows a specific diagnosis of cervical thymus and pathology correlation is not indicated.

It is a common practice at our center to do USG correlation when there is any ambiguity in the neck (including cervical thymus) on CT scan. We encountered some cases where the cervical component of thymus had a density different from the mediastinal thymus, but ultrasound (along with CT continuity with mediastinal thymus on sagittal/coronal images) confirmed that the cervical lesion was indeed a normal variant and not some small lymph node/other pathology. We did a retrospective study of cases of the cervical thymus in which USG correlation was available, to compare CT densities of cervical and mediastinal parts of thymus.

## Materials and methods

### Subjects

**Fig. 1:**
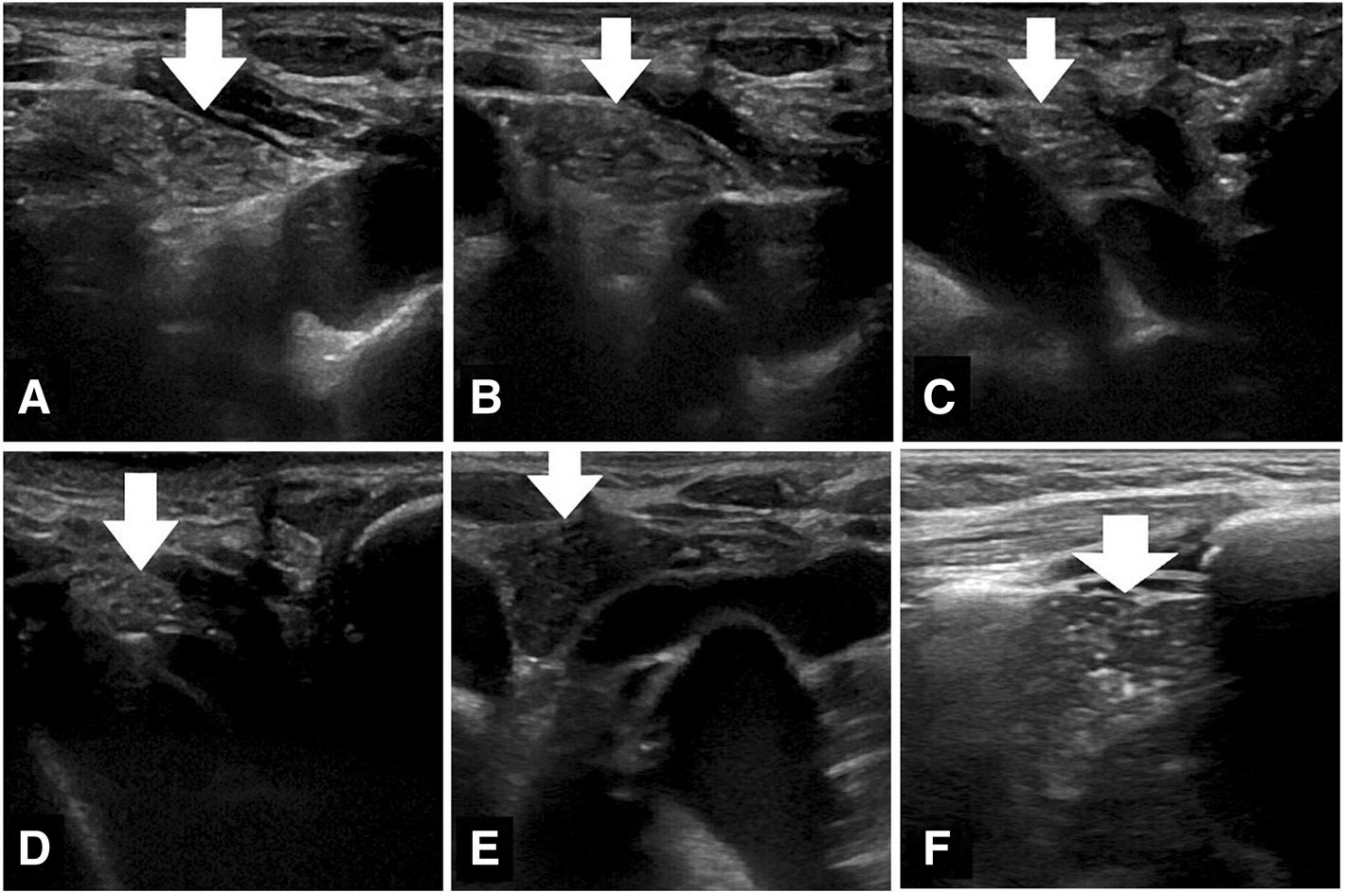
Transverse (axial) USG sections of thymus in lower neck and upper mediastinum. **a**–**d** Images of a 19-year-old male patient shows thymus (arrows) extending from neck to superior mediastinum. **e** Hypoechoiec thymus (arrow) in a 4-year-old female. **f** Hyperechoiec starry sky pattern of thymus (arrow) in an 8-year-old male

The institutional review board approved this retrospective study of existing imaging data, and written consent was waived. We retrospectively searched our USG and CT (PACS) records to identify patients in whom USG correlation (Fig. 1) had been performed to confirm cervical thymus, between May 2015 and May 2017. Inclusion criterion was a CT that included the lower neck (from the level inferior part of thyroid) and upper thorax (till main pulmonary artery). Exclusion criteria were mass lesions/lymph nodes in the lower neck, streak artifacts preventing clear evaluation of the lower neck.

### Imaging

CT was performed on a 16-channel scanner (LightSpeed; GE Healthcare, Milwaukee, WI) with variable milliampere and kilovolt (peak) of 120. Display FOV varied with patient size but was approximately 25 mm; coronal and sagittal reformats were used in this evaluation.

**Fig. 2:**
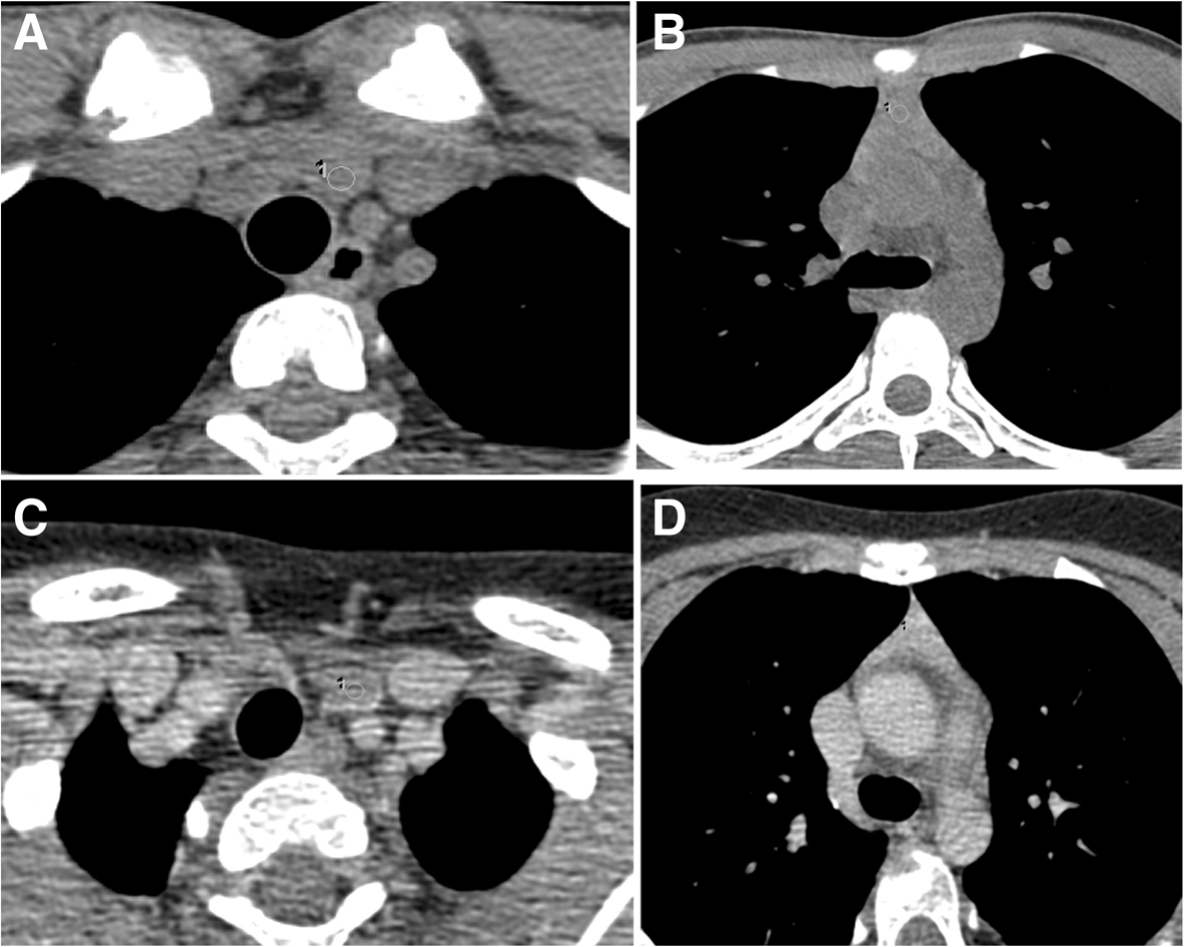
**a**, **b** Non-contrast CT images of neck and thorax of a 15-year-old male patient. **c**, **d** Contrast CT images of neck and thorax of a 10-year-old female patient. **a** Cervical thymus shows density of 35 HU on non-contrast CT. **b** Mediastinal thymus shows density of 61 HU on non-contrast CT. **c** Cervical thymus shows density of 46 HU on post-contrast CT. **d** Mediastinal thymus shows density of 100 HU on post-contrast CT

Cervical thymic tissue was defined as an oval focus of soft tissue above the level of sternal notch that had continuity with mediastinal thymus on axial/sagittal/coronal images. Each CT examination was evaluated by two radiologists (J.V. and A.S., with 17 and 11 years of post-PG experience, respectively) to determine densities of mediastinal and cervical components of thymus (Fig. 2) and the average values were recorded.

### Statistical tests

The difference in density of cervical and mediastinal parts of thymus was tested using paired *t* test. The comparison of density with age (groups) was tested using ANOVA test and the comparison of density with sex was tested using Student’s *t* test. A *p* value of .05 was chosen as the threshold for statistical significance. SPSS, Version 11 (IBM, Armonk, NY) was used for all statistical calculations.

## Results

The 22 studied patients included 10 males (45.5%) and 12 female patients (54.5%); age varied from 1 to 34 years (mean age 14); 19 patients had contrast-enhanced CTs and 3 patients had non-contrast CTs Additional file 1.

1. There is a difference between CT densities of cervical and mediastinal parts of thymus (*p* 0.001). Mean value of CT density of cervical thymus is lower than CT density of mediastinal thymus by ~ 25 units (Table 1) when 22 patients were considered together. Significant difference of mean value of CT density was also noted in the group of 19 post-contrast CTs (25.79; Table 2). Difference of mean value of CT density was also noted in the group of 3 non-contrast CTs (20; Table 3), though *p* value was higher due to the low sample size.
2. There is a moderate positive correlation between CT densities of cervical and mediastinal parts of thymus (correlation co-efficient 0.69). That is, if the CT density of mediastinal part of thymus is high, CT density of cervical thymus is also likely to be high; and vice versa.
3. CT densities of both cervical and mediastinal thymus were found to reduce with age, but the reduction was statistically significant only in the cervical thymus in this study (Table 4).
4. After adjusting for age, there is no difference demonstrated in densities (of both cervical and mediastinal thymus) between males and females.

**Table 1:** Comparison of CT densities of cervical and mediastinal parts of thymus by paired *t* test

		Mean	Std. deviation	Correlation	Mean difference	SD of mean diff	*p* value
Pair 1	CT density cervical	49.55	29.589	0.691	− 25.000	21.465	0.001
	CT density mediastinal	74.55	22.243

**Table 2:** Comparison of CT densities of cervical & mediastinal parts of thymus in 19 post-contrast CTs by Paired *t* test

C (*n* = 19)	Mean	Std. deviation	Mean difference (MD)	SD of MD	*p* value
CT density cervical	51.42	30.073	− 25.789	22.656	0.001
CT density mediastinal	77.21	19.412

**Table 3:** Comparison of CT densities of cervical & mediastinal parts of thymus in 3 plain (non-contrast) CTs by Paired *t* test

*p* (*n* = 3)	Mean	Std. deviation	Mean difference (MD)	SD of MD	*p* value
CT density cervical	37.67	28.431	− 20.000	13.229	0.120
CT density mediastinal	57.67	36.171

**Table 4:** Comparison of CT densities of cervical and mediastinal thymus with age by ANOVA test

		Frequency	Mean	Std. deviation	*p* value
CT density cervical	0–10 years	10	62.5	26.496	0.041
	11–20 years	7	49.86	21.177	
	> 20 years	5	23.2	32.507	
	Total	22	49.55	29.589	
CT density mediastinal	0–10 years	10	79.8	18.432	0.139
	11–20 years	7	79.43	8.619	
	> 20 years	5	57.2	35.138	
	Total	22	74.55	22.243	

## Discussion

Embryological pathway of thymic descent is from the angle of mandible to the superior mediastinum (along thymopharyngeal duct); hence, thymus can be found anywhere along this path. Continuity with mediastinal thymus distinguishes cervical component of thymus from ectopic thymic tissue. Cervical component of thymus may be present at all ages [1], though it is more commonly imaged in children and young adults [2] (frequency decreases with age).

On ultrasound, thymus of infants may be homogeneous, with echogenicity slightly less than liver and spleen [6] or have multiple linear or branching echogenic foci [7]. In older children, the classical ‘starry sky’ pattern may be seen due to the mixture of lymphoid tissue and fat; it has also been called ‘speckled appearance’ due to the hyperechoiec septae and hyperechoiec foci on a hypoechoiec background [4]. Continuity with rest of the (mediastinal) thymus [4], and the same USG characteristics as mediastinal thymus [4] may be noted. Since the thymus is very pliable, cardiac pulsations and respiratory movements may affect its shape, and this may be noticeable on real-time ultrasound [5, 8]. The characteristic sonologic features of cervical thymus obviate the need for biopsy/surgical removal [4, 5, 9].

Thymic size and density are affected by a number of factors. Thymic size decreases with age; acute size reduction may occur during stress (infection, neoplasm, surgery, radiotherapy, steroid administration, and chemotherapy); true hyperplasia occurs in recovery from recent stress [10]; when reactive (true) hyperplasia exceeds 50% of original thymic size, it is called rebound hyperplasia. Fatty change is associated with increasing age, cigarette smoking, high BMI, and male sex [11]. Post-contrast CT density of > 41.2 HU was found to be suggestive of lymphofollicular hyperplasia (seen in a variety of immunologically mediated diseases) rather than true hyperplasia in one study [12], but further studies are needed.

Our study demonstrates that CT density of cervical part of thymus may vary from that of mediastinal part of the thymus, with density of cervical thymus being lower than the density of mediastinal component. But densities of cervical and mediastinal parts of thymus show positive correlation. We did not study the inter-observer variation in CT density, based on the excellent intra- and inter-observer agreements noted previously [11, 13, 14]. Decrease in CT density of thymus with age, noted previously in mediastinal thymus [11], has been demonstrated in the cervical thymus in our study.

The difference in CT density between cervical and mediastinal parts of the thymus may be partially due to the non-involvement of the cervical thymus in thymic hyperplasia. Most of our oncology patients would have had stress (neoplasm, infection, surgery, chemotherapy, radiotherapy, steroid administration) and reactive thymic hyperplasia after recovery from stress.

Limitations of our study include the retrospective nature of the study and absence of histological correlation. All patients in the study were imaged for non-uniform clinical reasons. Sick patients may have a higher rate of thymic hyperplasia and hence increased the difference in densities between cervical and mediastinal components of the thymus, but this is acceptable because it mirrors the actual incidence that would be seen on medical imaging.

## Conclusion

CT densities of cervical and mediastinal components of thymus may vary, with density of cervical thymus being lower than the density of mediastinal component. When continuity with mediastinal thymus is seen on CT, diagnosis of the cervical thymus can be made even in the presence of different CT densities. Densities of cervical and mediastinal parts of thymus show positive correlation. Decrease in CT density of thymus with age, noted previously in the mediastinal thymus, has been demonstrated in cervical thymus also. Understanding these may help avoid confusion on CT and avoid the need for correlative USG, saving time and effort.

## Additional files

Additional file 1: **Table S1.** Patient data. (DOCX 20 kb)

## Data Availability

Attached as Additional file 1

## Abbreviations

CT: Computed tomography
USG: Ultrasonography
