# Supplementary material for: CT density of cervical thymus, in comparison with mediastinal thymus
Source: Insights Imaging. 2019 Sep 30;10:97. doi: 10.1186/s13244-019-0781-z (PMC6766458; doi:10.1186/s13244-019-0781-z)
Supplement: Supplementary file 1 — Table S1. Patient data. (DOCX 20 kb) [file 13244_2019_781_MOESM1_ESM.docx]

**Table S1:** Patient Data

| **Sl no:** | **SEX** | **AGE** | **contrast/plain CT** | **CT density cervical thymus** | **CT density mediastinal thymus** | **disease** |  |
| --- | --- | --- | --- | --- | --- | --- | --- |
| 1 | f | 34 | c | 5 | 66 | lymphoma |  |
| 2 | m | 16 | c | 77 | 70 | carcinoma thyroid |  |
| 3 | m | 15 | c | 74 | 78 | lymhoma |  |
| 4 | f | 8 | c | 77 | 83 | normal thymus mistaken for thyroid mass | histopathology proved thymus |
| 5 | m | 10 | c | 80 | 75 | lymphoma |  |
| 6 | m | 7 | c | 90 | 85 | lymphoma |  |
| 7 | m | 3 | c | 44 | 73 | lymphoma |  |
| 8 | m | 12 | c | 47 | 69 | lymphoma |  |
| 9 | f | 24 | c | 21 | 69 | lymphoma |  |
| 10 | f | 9 | c | 40 | 70 | Langerhans cell histiocytosis |  |
| 11 | f | 25 | c | 80 | 105 | carcinoma thyroid |  |
| 12 | f | 20 | c | 28 | 87 | carcinoma thyroid |  |
| 13 | m | 14 | p | 46 | 81 | osteosarcoma |  |
| 14 | f | 4 | c | 67 | 80 | neuroblastoma |  |
| 15 | m | 18 | c | 21 | 78 | lymphoma |  |
| 16 | m | 10 | c | 5 | 56 | neuroblastoma |  |
| 17 | m | 20 | c | 56 | 93 | lymphoma |  |
| 18 | f | 21 | c | 4 | 30 | duodenal PNET |  |
| 19 | f | 1 | c | 70 | 73 | angiosarcoma perineum |  |
| 20 | m | 8 | c | 91 | 127 | lymphoma |  |
| 21 | m | 21 | p | 6 | 16 | Ewings sarcoma |  |
| 22 | f | 9 | p | 61 | 76 | osteosarcoma |  |

Abbreviations: f = female; m = male; c = contrast CT study; p = plain (non-contrast) CT study; PNET = Primitive Neuroectodermal Tumour
